# Label-Free Proteomic Analysis of Molecular Effects of 2-Methoxy-1,4-naphthoquinone on *Penicillium italicum*

**DOI:** 10.3390/ijms20143459

**Published:** 2019-07-14

**Authors:** Meixia Guo, Xiaoyong Zhang, Meiying Li, Taotao Li, Xuewu Duan, Dandan Zhang, Lianmei Hu, Riming Huang

**Affiliations:** 1 Guangdong Provincial Key Laboratory of Food Quality and Safety, College of Food Science, South China Agricultural University, Guangzhou 510642, China; 2 Joint Laboratory of Guangdong Province and Hong Kong Region on Marine Bioresource Conservation and Exploitation, College of Marine Sciences, South China Agricultural University, Guangzhou 510642, China; 3 Guangdong Provincial Key Laboratory of Applied Botany, South China Botanical Garden, Chinese Academy of Sciences, Guangzhou 510650, China; 4 College of Veterinary Medicine, South China Agricultural University, Guangzhou 510642, China

**Keywords:** *Penicillium italicum*, 2-methoxy-1,4-naphthoquinone, proteomics, mechanism, label-free quantitative

## Abstract

*Penicillium italicum* is the principal pathogen causing blue mold of citrus. Searching for novel antifungal agents is an important aspect of the post-harvest citrus industry because of the lack of higher effective and low toxic antifungal agents. Herein, the effects of 2-methoxy-1,4-naphthoquinone (MNQ) on *P. italicum* and its mechanism were carried out by a series of methods. MNQ had a significant anti-*P. italicum* effect with an MIC value of 5.0 µg/mL. The label-free protein profiling under different MNQ conditions identified a total of 3037 proteins in the control group and the treatment group. Among them, there were 129 differentially expressed proteins (DEPs, up-regulated > 2.0-fold or down-regulated < 0.5-fold, *p* < 0.05), 19 up-regulated proteins, 26 down-regulated proteins, and 67 proteins that were specific for the treatment group and another 17 proteins that were specific for the control group. Of these, 83 proteins were sub-categorized into 23 hierarchically-structured GO classifications. Most of the identified DEPs were involved in molecular function (47%), meanwhile 27% DEPs were involved in the cellular component and 26% DEPs were involved in the biological process. Twenty-eight proteins identified for differential metabolic pathways by KEGG were sub-categorized into 60 classifications. Functional characterization by GO and KEGG enrichment results suggests that the DEPs are mainly related to energy generation (mitochondrial carrier protein, glycoside hydrolase, acyl-CoA dehydrogenase, and ribulose-phosphate 3-epimerase), NADPH supply (enolase, pyruvate carboxylase), oxidative stress (catalase, glutathione synthetase), and pentose phosphate pathway (ribulose-phosphate 3-epimerase and xylulose 5-phosphate). Three of the down-regulated proteins selected randomly the nitro-reductase family protein, mono-oxygenase, and cytochrome P450 were verified using parallel reaction monitoring. These findings illustrated that MNQ may inhibit *P. italicum* by disrupting the metabolic processes, especially in energy metabolism and stimulus response that are both critical for the growth of the fungus. In conclusion, based on the molecular mechanisms, MNQ can be developed as a potential anti-fungi agent against *P. italicum*.

## 1. Introduction

Plant pathogenic fungi negatively affects a large number of important fruits during the growing season and throughout post-harvest storage that cause serious losses of yield and quality for a number of fruits worldwide [1,2]. For instance, 10% to 30% of citrus fruit loss is caused by blue mold disease throughout the world [3]. One of the most usual post-harvest fungal diseases of citrus fruits is blue mold caused by *Penicillium italicum*. Agricultural practices and biological measures only provided limited relief against this disease. Thus, a large number of commercial fungicides were used to control the blue mold [4]. Application of numerous chemical synthetic fungicides is the major method to control *P. italicum* of citrus. However, the application of synthetic fungicides has been increasingly restricted because of a series of severe issues that arose from these chemical synthetic fungicides, such as residues of toxic compounds, environmental pollution, pathogen resistant development, and health hazard problems [4]. Compared to chemical synthetic antifungal agents, natural compounds are generally found to be safe due to less toxicity or no toxicity. It is, therefore, urgent to explore novel effective and non-toxic or less toxic antifungal agents with new modes of action. The numbers of research studies have reported the antifungal effects of natural antifungal compounds for restraining postharvest blue mold caused by *P. italicum* [3,5,6,7,8].

**Figure 1:**
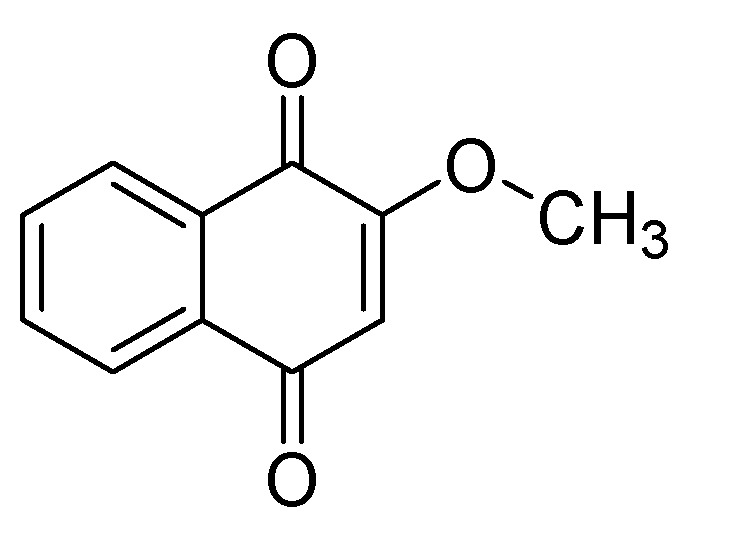
2-Methoxy-1,4-naphthoquinone (MNQ).

A 2-methoxy-1,4-naphthoquinone (MNQ) (Figure 1) is a naturally occurring phytochemical isolated from a traditional Chinese medicinal plant, *Impatiens balsamina* Linn [9]. Previous studies showed that MNQ possessed diverse biological activities including anti-cancer [10], anti-metastatic [11] , and anti-bacterial [12] properties. However, there is still a lack of more data about the antifungal activities of this 1,4-naphthoquinone derivative [10,13]. Nonetheless, our present investigation indicated that the MNQ had a significant anti-fungal activity against *P*. *italicum* as a promising anti-fungal precursor. However, the possible antifungal molecular mechanisms of MNQ *P. italicum* have not been investigated yet. Thus, the possible mechanisms involving changes of morphological, changes of proteins and pathways, and the energy deficit in *P. italicum* should be further investigated because the biomolecules functionally govern cellular processes and, ultimately, dictate the biological phenotypes, proteins that are primary targets of drug discovery [11], and the differentially expressed proteins (DEPs). These offer clues on the proteins and pathways affected by a drug treatment [14].

In this study, we aimed to investigate the modulatory effects of MNQ toward the *P. italicum* proteome by using a label-free quantitative proteomic approach to identify proteins with significantly changed expression profiles, to confirm their functions in the MNQ-treated *P. italicum*. Moreover, three selected proteins related to oxidative stress and polycyclic aromatic hydrocarbon degradation were also confirmed using parallel reaction monitoring (PRM) analysis. Ultimately, these findings will contribute to our further understanding of the possible new molecular action of MNQ against *P. italicum*.

## 2. Results

### 2.1. Effects of MNQ on P. italicum

**Figure 2:**
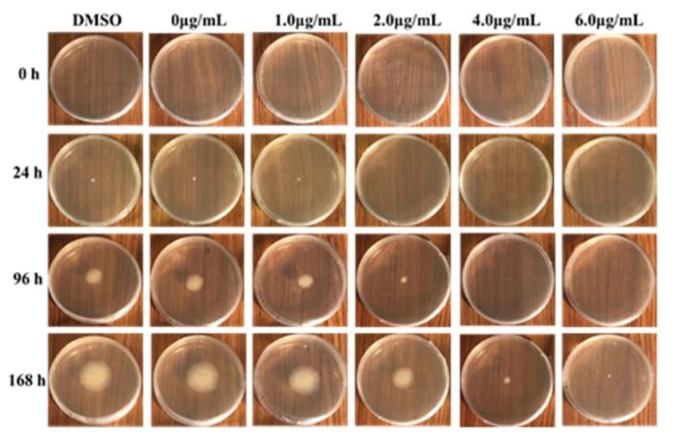
Inhibition of mycelial growth of *P. italicum* by MNQ.

The inhibitory effect of MNQ on *P. italicum* was carried out using a mycelial growth rate method. As shown in Table 1, MNQ at 1.0 μg/mL showed a blank antifungal activity against *P. italicum*. When the concentrations of MNQ are at 2.0, 4.0, and 6.0 μg/mL, MNQ displayed a high anti-fungal activity against *P. italicum*. Among them, MNQ at 6.0 μg/mL exhibited a stronger antifungal activity against *P. italicum*. As depicted in Figure 2, *P. italicum* mycelial growth showed a declining tendency with the increasing concentrations of MNQ. The MIC value was defined as the lowest MNQ concentration that completely inhibited the growth of *P. italicum* after 48 hours of incubation at 28 °C [15]. MNQ showed a significant anti-*P. italicum* effect with an MIC value of 5.0 µg/mL (Figure S1).

### 2.2. Light Microscopy Observation

**Figure 3:**
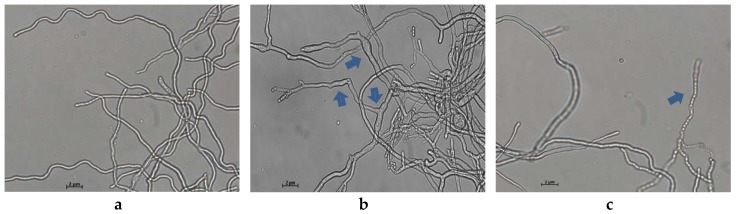
Light microscopy observation of *P. italicum* mycelial morphology (96 h). (**a**) *P. italicum* in the control group, (**b**) and (**c**) *P. italicum* in the treatment group (magnification is 400 times).

Light microscopy of the control *P. italicum* mycelia grown on PDB showed a normal, filamentous, and homogeneous mycelial morphology (Figure 3a). By contrast, the mycelia treated with 2.0 μg/mL of MNQ showed a massive distortion and abnormal enlargement of the growing point. Meanwhile, Figure 3b,c showed that the MNQ-treated mycelia significantly altered the mycelial morphology of *P. italicum*, including breakage and malformation (highlighted with blue arrows). Clearly, MNQ treatment resulted in the damage to *P. italicum* mycelia (magnification is 400 times).

### 2.3. Label-Free Proteomic Based Protein Identification in the Treatment Group and Control Group

**Figure 4:**
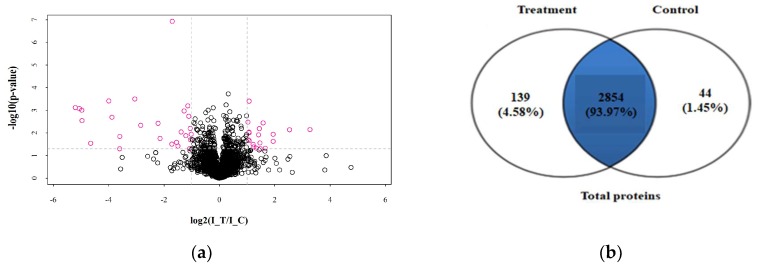
DEPs in different groups. (**a**) Volcano plot of DEPs, I-T was the treatment group. I-C was the control group. (**b**) Venn diagram of proteins in different groups.

**Figure 5:**
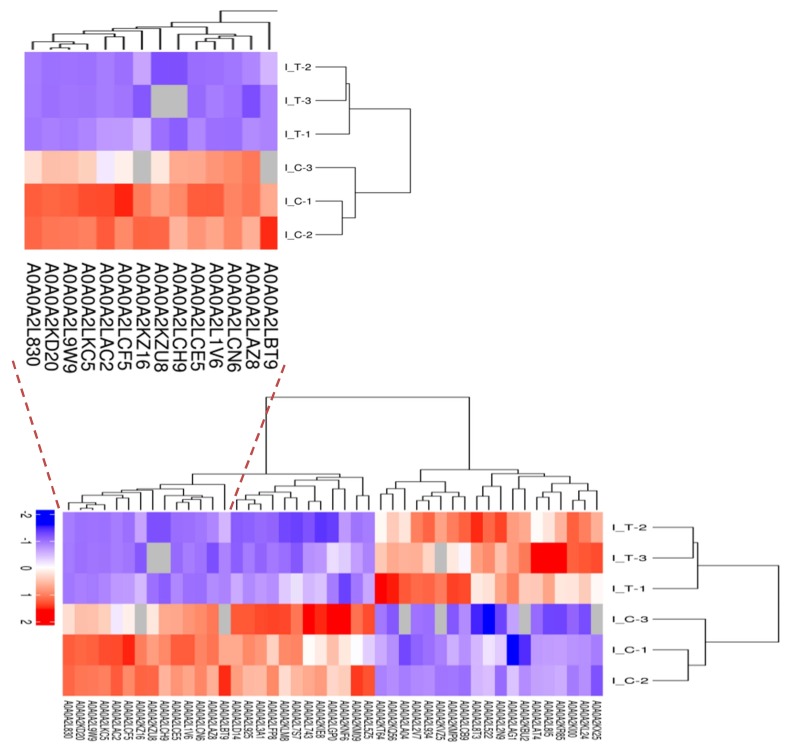
Hierarchical cluster analysis was conducted for all the DEPs. I-T was the treatment group. I-C was the control group. The numbers were the protein ID and could search in Table S1.

Quantitative proteomic analysis of mycelia samples from the treatment group and control group was performed using the label-free proteomic analysis method. In total, 3037 proteins were identified with a 1% false discovery rate (FDR) (Table S1). Figure 4 shows the total numbers of differentially expressed proteins in the treatment group and the control group. Figure 4a shows the volcano plot for the differentially expressed proteins (DEPs) in the treatment group and the DEPs (up-regulated > 2.0-fold or down-regulated < 0.5-fold, *p* < 0.05) highlight in a pink color. All the DEPs are listed in Table 2. The Venn diagram in Figure 4b shows the number of proteins that are unique to the treatment group or the control group. As shown in Figure 4b, there were 2854 proteins both expressed in the treatment group and the control group, 139 proteins were unique to the treatment group when compared to 44 for the control group. A total of 129 proteins showed significant changes in expression. Compared with the control group, 45 proteins (19 up-regulated and 26 down-regulated) were significantly changed in abundance in the treatment group (Table 3). Meanwhile, 67 newly arising proteins were identified in the treatment group, but not found in the control group, and 17 proteins were undetectable in the treatment group while observed in the control group. Hierarchical cluster analysis was performed for all the DEPs for the treatment group and the control group (Figure 5). As shown in Figure 5, it was easy to note that the differentially expressed proteins in the treatment group did not cluster together with the one in the control group.

### 2.4. Functional Annotation of Proteins

**Figure 6:**
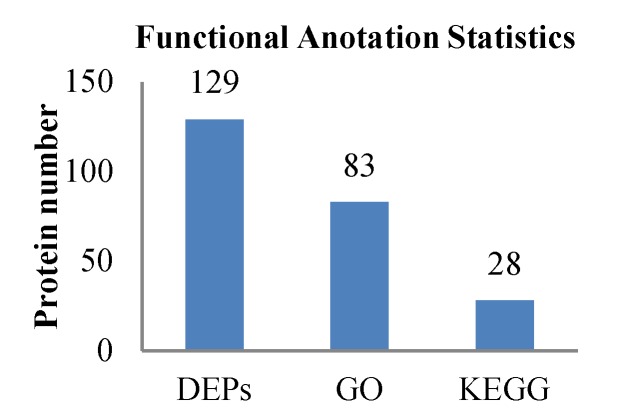
Statistical results of different functional annotations.

To deduce the functionality and biological processes associated with the identified DEPs in the treatment group and control group, gene ontology (GO) analysis and Kyoto Encyclopedia of Genes and Genomes (KEGG) enrichments were performed. The GO and KEGG annotation of the identified proteins were carried out to comprehensively reflect the biological functions and significance of these proteins in various life activities. Functional annotation of all proteins obtained from the treatment group and control group revealed a sum of 129 differential proteins. Of these, 83 proteins were sub-categorized into 23 hierarchically-structured GO classifications. Twenty-eight proteins identified for differential metabolic pathways by KEGG were sub-categorized into 60 classifications (Figure 6).

#### 2.4.1. Gene Ontology (GO) Annotation

**Figure 7:**
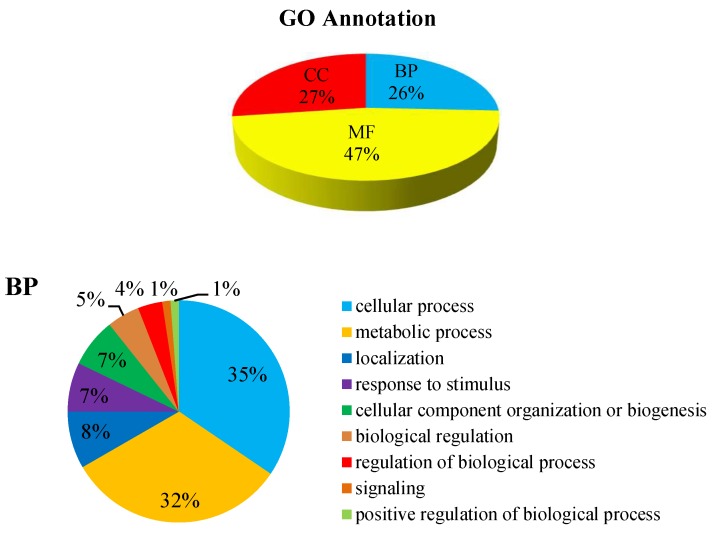

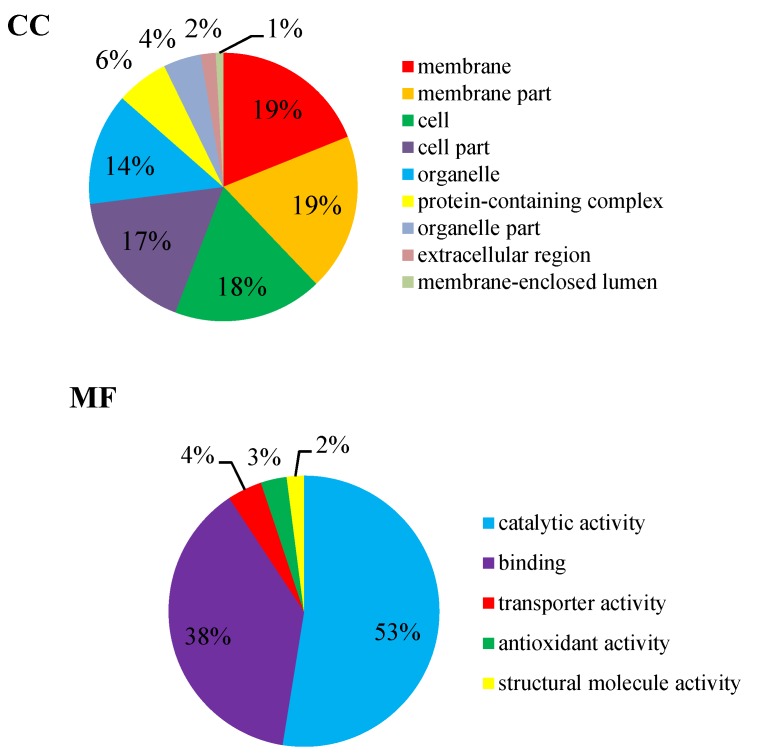
GO annotation for the DEPs.

GO annotation gives a priority for a systematical analysis of the protein properties of targeted proteins [16]. In order to get a detailed description, GO was further categorized into three components e.g., biological process (BP), cellular component (CC), and molecular function (MF). The GO analysis revealed that the identified DEPs were associated with different molecular and biological processes. Most of the identified DEPs were involved in molecular function (47%). Meanwhile 27% DEPs were involved in the cellular component and 26% DEPs were involved in the biological process (Figure 7). The biological process was found to be highly enriched in the cellular process (35%) and the metabolic process (32%). At the cellular component level, the identified DEPs were linked to the membrane (19%), membrane part (19%), cell (18%), cell part (17%), and organelle (14%). Similarly, catalytic activity (53%) and binding (38%) were most significantly expressed in the molecular function. Altogether, these results suggest that many proteins in *P. italicum* under MNQ treated might be involved in different metabolic and cellular processes and localize in different cell parts and organelles.

#### 2.4.2. Metabolic Pathway Annotation

The DEPs coordinate with each other to express their biological behavior. Thus, the pathway-based annotation broadens further exploration of their biological functions. The pathway analysis can determine important biochemical, ametabolic, and signaling pathways regulated by proteins. The KEGG database results indicated that the differential proteins participated in a total of 60 signaling pathways (Table 4). From these, the top 10 metabolic pathways were dioxin degradation, polycyclic aromatic hydrocarbon degradation, linoleic acid metabolism, betalain biosynthesis, sphingolipid metabolism, mineral absorption, longevity regulating pathway, pentose and glucuronate interconversions, MAPK signaling pathway–yeast, and the mRNA surveillance pathway (Figure S2).

### 2.5. PRM Verification of DEPs

The PRM assay was applied to validate the selected proteins obtained in the label-free quantitative proteomics analysis. Only proteins that possessed unique peptide sequences were selected for PRM analysis because the signature peptides for target proteins must exhibit uniqueness [17]. Three of the proteins were selected at random for further verification. The PRM results showed that the abundance of nitroreductase family protein (from *Metarhizium brunneum*, identity 90.4%), cytochrome P450, and monooxygenase changed in a consistent way with the proteomics results (Table 5). In general, three of the candidate proteins detected by PRM had changes consistent with the proteomics results.

## 3. Discussion

### 3.1. Effects of MNQ on the Mycelial Growth and Morphology of P. italicum

For a pathogen, a drug may prove fatal depending on the level of drug exposure (time and concentration). Our present study showed that the mycelial growths of *P*. *italicum* were decreased in a dose-dependent manner. MNQ treatment significantly reduced the mycelial growth of *P*. *italicum* (Table 1). However, microorganisms are extremely adaptable because their lifestyles require the swift phenotypic adaptation with a variety of changing environmental factors [18]. If the pathogenic fungi are not inhibited or killed by drug exposure, its will render the organism less susceptible to drug toxicity because the drug challenge may be met by adaptive responses [19].

Microscopic analyses showed a clear difference between the treatment group and control group mycelia of *P. italicum*. The MNQ (2.0 μg/mL) treated mycelia were inflated at the growing point. Meanwhile, those mycelia exposed to MNQ treatment were malformation and collapsed. The present study revealed that the prominence of mycelia decreased the mycelia grew, and the changes of mycelia may have been attributed to amplifying membrane permeability. It would result in the swelling of the mycelial tips, collapse of the cell wall, leakage of cell constituents, and, ultimately, mycelia growth was restricted or cell death (Figure 2 and Figure 3). In a word, microscopy observation showed that MNQ resulted in an irregular variation of *P. italicum* mycelia, which indicates that the inhibitory effect of MNQ on *P. italicum* could be linked with membrane permeability changes modulated by the membrane lipid composition. Ergosterol is one of the main lipid molecules in the fungi cell membrane. It regulates membrane fluidity, permeability, and the activity of many membrane-bound enzymes [20]. Furthermore, the inhibition on ergosterol synthesis may result in changes of the cell wall structure and morphology [21]. Under the stress of the MNQ, it is more difficult for *P. italicum* to maintain cell wall integrity or to produce a new cell wall. The change of mycelial morphology suggested that MNQ can play an important role in destroying *P. italicum* viability.

### 3.2. Proteins Related to Energy Generation and the NADPH Supply and Other Functions

Proteomics results suggested that MNQ elicited a cellular response and some peculiar mechanisms that were very different between the treatment group and control group due to MNQ exposure is an environmental stress to a pathogen who must raise the response [19]. A fitting response to these adversities that might be caused by MNQ includes physiological and developmental changes, reprogramming of the resistance gene or proteins, the affection of energy generation, and the NADPH supply. Proteomics analysis revealed that MNQ treatment significantly up-regulated four energy generation related proteins including the mitochondrial carrier protein, glycoside hydrolase, acyl-CoA dehydrogenase, and ribulose-phosphate 3-epimerase (Table 2). The results showed that the mitochondrial carrier protein increased by 2.51-fold in the treatment group. The increase of the mitochondrial carrier protein may lead to accelerated material transport, which suggests that the biosynthesis and degradation rate in the mitochondria of cells were accelerated [22] because mitochondria play an important metabolic role in eukaryotes that generate the energy in the form of ATP [23]. In addition, the acyl-CoA dehydrogenase (ACAD) can catalyze α, β-acyl-CoA esters dehydrogenation in the amino acid, and fatty acid catabolism [24]. Both acyl-CoA oxidases and ACAD that is bona fide peroxisomal proteins in fungi are the basic enzymatic repertoire of peroxisomes [25]. The increase of ACAD indicated that fatty acid and amino acid catabolism were active in the treatment group.

Meanwhile, there were five proteins up-regulated including enolase, pyruvate carboxylase, ATP-dependent 6-phosphofructokinase, malate synthase, and phosphoenolpyruvate carboxykinase that play a role in the NADPH supply (Table S1). Enolase, as a significant metabolic intermediate for ATP and NADH production, is a ubiquitous glycolytic enzyme involved in the glycolysis and gluconeogenesis pathways, and it catalyzes the reversible dehydration conversion of 2-phosphoglyceric acid into phosphoenolpyruvate [26]. In our study, the up-regulated enolase may play an important role in energy generation and cell apoptosis. NADPH plays a vital role in fatty acid synthesis. The change of NADPH may affect the cell integrity because phospholipids are the main content factors of the cell membrane [27]. Some of these enzymes including malic enzyme (ME) and 6-phosphogluconate dehydrogenase (6PGDH) also play important roles in generating NADPH/NADH. ME is a primary source of NADPH for de novo lipid biosynthesis and desaturation. Meanwhile, 6PGDH is one of the enzymes that catalyzes reactions generating NADPH in the oxidative pentose phosphate pathway [28]. We found that ME and 6PGDH increased 1.03-fold and 1.15-fold, but the expression difference was not consistent with the significance criteria (Table S1). It is reported that NADPH could prevent oxidative stress in the cell. For example, NADPH reduces glutathione through glutathione reductase because NADPH could convert H_2_O_2_ into H_2_O by glutathione peroxidase. If NADPH is absent, the H_2_O_2_ would be converted to hydroxyl-free radicals to attack the cell [27]. NADPH in the *P. italicum* might increase the fatty acid synthesis and oxidation resistance that may improve the defense during treated MNQ.

The stress condition tends to disrupt cell and organelle membranes and cause an ionic imbalance that stimulates the generation of reactive oxygen species (ROS) [29]. The generation of ROS leads to apoptosis [30]. Previous studies indicated that the antioxidant proteins were the first cellular defense against the oxidative stress. Glutathione transferase and dioxygenases may be related to the response of the fungus to oxidative stress caused by an ROS increase and ATP production [31]. Our proteomics analysis revealed that MNQ treatment up-regulated seven oxidative stress related proteins including catalase (CAT), acyl transferase, glutathione synthetase carboxymuconolactone decarboxylase, tRNA-dihydrouridine (47) synthase [NAD(P)(+)], polyketide synthase, and aldolase-type TIM barrel (Table 2). Antioxidant enzymes such as CAT was up-regulated when exposed to MNQ, which shows its important role in detoxification of ROS. In addition, antioxidant enzymes against ROS, such as peroxidase and alcohol dehydrogenase were also up-regulated, but the expression difference did not meet the significance criteria when exposed to MNQ stress as compared to the control group (Table S1). Glutathione in fungi and some bacteria is an important antioxidant with a capability of preventing damage to important cellular constituents caused by ROS [32]. Glutathione as a substrate, is also contributed to the regeneration of other antioxidants [33]. Glutathione biosynthesis involves two sequential ATP dependent reactions, and is mediated by two enzymes γ-glutamylcysteine synthetase and glutathione synthetase (GS). Energy generation enhanced and GS increased 1.09-fold meant that glutathione increased in the treatment group. We hypothesized that the change of energy generation, NADPH supply, and oxidative stress affected the cell growth and mycelial morphology that was also confirmed by the *P. italicum* mycelial growth curve and microscopic analyses (Figure 2 and Figure 4).

Among the pentose phosphate pathway (PPP), two enzymes called ribulose-phosphate 3-epimerase (RPE) and xylulose 5-phosphate were up-regulated. RPE can play a vital role in the development of a pool of NADPH, in the PPP that can convert monosaccharides, such as glucose, into the nucleotide precursor of pentose sugars. In addition, RPE can convert the ribulose-5-phosphate into xylulose 5-phosphate in the Calvin cycle [34]. RPE was identified with reversibly oxidized cysteines, which was another enzyme that uses ribulose-5-phosphate as the substrate. We found other published sources that reported the rpe (ribulose phosphate 3-epimerase gene)-disrupted mutant resulted in low cell growth and low inosine production on glucose and on gluconate [35]. Furthermore, RPE was up-regulated significantly, which suggests that PPP was significantly active in the treatment group. This pathway has been suggested as a drug target in trypanosomes [34].

According to the above analysis, we speculated that MNQ inhibited the growth of *P. italicum* by affecting the generation of energy and NADPH, which, thereby, affects the synthesis of fatty acids and amino acids. Meanwhile, PPP may be the target of MNQ for *P. italicum*, but the actual situation still needs to be further investigated.

## 4. Materials and Methods 

### 4.1. Microorganism and Medium

*P. italicum* was provided by the South China Botanical Garden, Chinese Academy of Sciences (Guangzhou, China). *P. italicum* was cultured on potato dextrose agar (PDA, potato 200 g/L, agar 20 g/L, and dextrose 20 g/L) or potato dextrose broth (PDB, potato 200 g/L and dextrose 20 g/L).

### 4.2. MNQ Inhibition of P. italicum

#### 4.2.1. Antifungal Assay

The effects of MNQ on mycelial growth were measured as described by Myresiotis et al. [36]. MNQ was suspended in DMSO and the stock solution of MNQ (10,000 μg/mL) was added to the PDA media (50 mL) contained in conical flasks to obtain the desired concentrations of MNQ (0, 1.0, 2.0, 4.0, and 6.0 μg/mL) in the media. The medium was poured into a set of three Petri dishes (90 mm in diameter) under aseptic conditions. Then the plates were placed under UV light for solidification of the media. Meanwhile, we transferred the mycelia of *P. italicum* grown on PDA (about 7 days) to PDB in order to obtain conidial suspensions (about 1 × 10^5^ CFU/mL). After that, 1 μL of *P. italicum* (1 × 10^5^ CFU/mL conidial suspensions) were transferred to the center of the PDA plates. PDA media amended with DMSO (20 μL) was used as the control. Each treatment in the experiment had three replicate plates, and was repeated twice. All cultivations were carried out at 28 °C. The inhibition of mycelial growth rate (%) was calculated using the following formula [37].

I (%) = (C−T) / C × 100%(1)

where C and T were the mean mycelium colony diameters of the control and the treatment and I was the inhibition of the mycelial growth rate (%).

#### 4.2.2. Minimum Inhibitory Concentration (MIC) Tests

The effects of MNQ on *P. italicum* were performed with a minimum inhibitory concentration (MIC) value [38]. MNQ was tested against *P. italicum* at different concentrations of 40.0, 20.0, 10.0, 5.0, 2.5, 1.25, 0.625, and 0.3125 μg/mL. The stock solution of MNQ (10,000 μg/mL) was first diluted to the highest concentration (40 μg/mL), and then serial two-fold dilution was made in a concentration range from 40.0 to 0.3125 μg/mL. The solutions poured into 50 mL conical flasks containing melted PDA medium. Then the medium was poured into a set of three Petri dishes (60 mm in diameter) under aseptic conditions. The plates were placed under UV light for solidification of the media. After solidification, 20 μL of conidial suspensions (about 1 × 10^5^ CFU/mL) were then smeared evenly onto the surface of the plates, aseptically. Lastly, all the culture plates were incubated at 28 °C for 48 h. Each treatment had three replicate plates, and the experiment was repeated twice.

### 4.3. Cell Culture and MNQ Treatment

Two milliliters of the conidial suspensions (about 1 × 10^5^ CFU/mL) were added into a 1000 mL conical flask containing 200 mL of PDB. Then all the cultivations were carried out on a rotary shaker at 140 rpm at 28 °C. After two days of incubation, 40 µL of MNQ stock solution (10,000 μg/mL) was added to the three bottles of cultivations as the treatment group (2 µg/mL of MNQ), aseptically. The cultivations without dosing any MNQ were considered to be the control group. Then, all cultivations were carried out on a rotary shaker at 140 rpm at 28 °C for another 2 days. For the treatment group, 2.0 μg/mL of MNQ was used in this work, and, without dosing, any MNQ was considered to be the control group. Three biological replicates were carried out for each condition. In this cases, sub-MIC doses of MNQ were applied to *P. italicum*, in order to impose stress to the cells but not induce non-specific effects associated with cell death [15]. The *P. italicum* mycelia were harvested after four days of cultivation. The mycelia were collected by centrifugation after being washed in three 5-minute steps with 0.1 M PBS (pH 7.4). Part of the mycelia is used for microscopic observation and part of it is used for proteomics analysis.

### 4.4. Label-Free Quantitative Proteomics

#### 4.4.1. Sample Preparation

The mycelia were obtained through centrifugation at 5000 g for 3 min, and then washed with PBS buffer three times. The cell pellets were suspended on ice in 200 μL of lysis buffer (4% SDS, 100 mM DTT, 150 mM TrisHCl, pH 8.0). Cells were disrupted by a homogenizer agitated (Fastprep-24^®^, MP Biomedical, USA), and the lysates were boiled for 5 min. The samples were further homogenized using ultra-sonication, and then boiled for another 5 min. Undissolved cellular debris were removed by centrifugation at 14,000 rpm for 15 min and the supernatants were collected for further investigation and quantified with a BCA Protein Assay Kit (Bio-Rad, Hercules, CA, USA).

#### 4.4.2. Protein Digestion

Digestion of protein (250 μg for each sample) was performed according to the FASP procedure [39]. In brief, the detergent, DTT, and low-molecular-weight constituents were removed using a 200 μL UA buffer (8 M Urea, 150 mM Tris-HCl pH 8.0) by repeated ultra-filtration (Microcon units, 30 kDa) facilitated by centrifugation. In order to reduce cysteine residues, 100 μL 0.05 M iodoacetamide added in UA buffer was to block reduced cysteine residues. These samples were incubated in the dark within 20 min. The filter was washed with a 100 μL UA buffer three times and then with a 100 μL 25 mM NH_4_HCO_3_ two times. The resulting proteins were digested using 3 μg trypsin (Promega, Madison, WI, USA) in 40 μL 25 mM NH_4_HCO_3_ overnight at 37 °C, and the resulting peptides were collected as a filtrate. The peptide content was estimated by a UV light spectral density of 280 nm.

#### 4.4.3. Liquid Chromatography (LC)-Electrospray Ionization (ESI) Tandem MS (MS/MS) Analysis

The peptide in each sample was desalted on C18 Cartridges (Sigma, St. Louis, MO, USA). It was then concentrated by a vacuum centrifugation and was reconstituted in 40 µL of 0.1% (*v/v*) trifluoroacetic acid. MS experiments were performed on a Q Exactive mass spectrometer coupled to an Easy nLC (ThermoFisher Scientific, Waltham, MA, USA). An aliquot comprising 5 μg peptide was loaded onto a C18-reversed phase column (Thermo Scientific Easy Column, 10 cm long, 75 μm inner diameter, 3 μm resin) in buffer A (2% acetonitrile and 0.1% formic acid) and was separated with a linear gradient of buffer B (80% acetonitrile and 0.1% Formic acid) at a flow rate of 250 nL/min controlled by an IntelliFlow technology over 120 min. A data-dependent top 10 method dynamically choosing the most abundant precursor ions from the survey scan (300–1800 m/z) for HCD fragmentation was carried out to obtain the MS data. Determination of the target value is on the basis of the predictive Automatic Gain Control (pAGC). Each sample was carried out in MS experiments three times.

#### 4.4.4. Sequence Database Searching and Data Analysis

The MS data were searched against the UniProtKB *P. italicum* database and were analyzed using a MaxQuant software (version 1.3.0.5). An initial search was set at a precursor mass window of 6 ppm. An enzymatic cleavage rule of Trypsin/P and a maximum of two missed cleavage sites were perform in this research. Cysteines carbamidomethylation was defined as fixed modification. Meanwhile, protein N-terminal acetylation and methionine oxidation were defined as variable modifications. The cutoff of global FDR for peptide and protein identification was set to 0.01. Label-free quantification was performed in MaxQuant, as preceding studies reported [40]. Protein abundance was calculated on the basis of the normalized spectral protein intensity (LFQ intensity). The fellow data of the target proteins were retrieved from the UniProtKB database in batches. Each query sequence was retrieved and loaded into Blast2GO (version 2.7.2) for GO annotation and KEGG pathway analysis.

The protein sequences of the DEPs were in batches retrieved in the FASTA format from UniProtKB database. The retrieved sequences were searched from the SwissProt database using the NCBI BLAST+ client software to find homologue sequences from which the functional annotation can be transferred to the studied sequences. The top 10 blast hits in each query sequence with E-value less than 1e^−3^ were retrieved and loaded into Blast2GO [41] for GO mapping and annotation. An annotation configuration with an E-value filter of 1e^−6^, default gradual EC weights, a GO weight of 5, and an annotation cutoff of 75 were chosen. The unannotated sequences were then reannotated with more permissive parameters. Then, we used the looser parameters to reannotate the unannotated sequence. Lastly, the unannotated sequences and sequences without BLAST hits were then selected to go through an InterProScan [42] against EBI databases to retrieve functional annotations of protein and merge the InterProScan GO terms to the annotation set. As for the KEGG pathway analysis, the FASTA protein sequences of DEPs were blasted against the online KEGG database (http://geneontology.org/) to retrieve their KOs and were, subsequently, mapped to pathways in KEGG [43].

### 4.5. Parallel Reaction Monitoring (PRM) Analysis

In order to confirm the protein expression levels identified by label-free quantitative proteomics analysis, we further quantified the expression levels of selected proteins. Based on femtomole peptide/microgram total protein, samples were added with known amounts of each standard to determine the number of respective proteins in the sample. We used parallel reaction monitoring (PRM) to analyzed samples [44,45]. All fragment ions were quantified in the orbitrap after each precursor ion (light and heavy masses) was selected by the fragmented quadrupole [46]. Skyline (MacCoss Lab Software version 3.1) was used for data analysis to estimate peptide signal intensity. According to the ratio of the heavy peptide standards that were added in the known quantity, peptide concentration was calculated. Ion activation/dissociation was acted at a normalized collision energy of 27 in the HCD collision cell [47]. Skyline were used to analyzed the raw data where signal intensities for individual peptide sequences for each of the significantly changed proteins were quantified relative to every sample and normalized to a standard reference.

### 4.6. Statistical Analysis

Statistical analysis was performed with a one-way ANOVA in Origin 6.1 software. The statistical analysis (Student’s t-test and G-test) was conducted by PepC44 on two conditions: the treatment group and the control group. The DEPs were filtered by the following cutoff: up-regulated > 2.0-fold or down-regulated < 0.5-fold, *p* < 0.05.

## 5. Conclusions

Overall, our present investigation revealed that MNQ had a significant anti-*P. italicum* activity with a MIC value of 5.0 µg/mL. The label-free protein profiling under different MNQ conditions identified a total of 3037 proteins in the control group and the treatment group. Among them, there were 129 DEPs, including 19 up-regulated proteins, 26 down-regulated proteins, and 67 proteins that were specific for the treatment group and another 17 proteins that were specific for the control group. Functional characterization by GO and KEGG enrichment results suggests that the DEPs are mainly related to energy generation, the NADPH supply, oxidative stress, and PPP. The PRM assay confirmed that the expression of the nitroreductase family protein, monooxygenase, and cytochrome P450 are significantly down-regulated in the *P. italicum* with MNQ treatment. Based on the microscopic observation and the proteomics results mentioned above, we speculated that the proteins associated with energy production, NADPH supply, and other functions in *P. italicum* were altered to reduce the damage caused by MNQ. Thus, these findings will contribute to further understanding of the possible new molecular action of MNQ against *P. italicum*.

## Figures and Tables

**Table 1:** Effect of different MNQ concentrations on mycelial growth of *P. italicum* ^1^.

Concentration (μg/mL)	Mycelial Diameter (mm)	I (%)
CK	33.83 ± 0.54 ^a^	--
0.0	32.67 ± 0.56 ^a^	3.45 ± 1.65 ^c^
1.0	30.83 ± 0.54 ^a^	8.87 ± 1.60 ^c^
2.0	17.33 ± 2.30 ^b^	48.77 ± 6.81 ^b^
4.0	2.33 ± 0.80 ^c^	93.10 ± 2.37 ^a^
6.0	0.83 ± 0.17 ^c^	97.54 ± 0.49 ^a^

^1^ Mycelial growth was measured after incubation at 28 °C for seven days. The date presented are the means ± S.E. (*n* = 6). ^a–c^ The column with different lowercase letters between different concentrations indicates significant differences, according to Duncan’s test (*p* < 0.05).

**Table 2:** The DEPs in different groups.

Protein	Protein Name	Gene Name	Fold Changes ^1^	GO Category ^2^
A0A0A2KI00	Uncharacterized protein	PITC_013530	9.66	MF
A0A0A2KQ56	Uncharacterized protein	PITC_017440	5.80	
A0A0A2LA04	Ribulose-phosphate 3-epimerase	PITC_011800	3.84	BP, MF
A0A0A2KBU2	Hydrophobin	PITC_015600	3.83	MF, CC
A0A0A2KT64	Uncharacterized protein	PITC_065480	3.15	
A0A0A2KL24	Uncharacterized protein	PITC_064010	2.99	
A0A0A2LAT4	Uncharacterized protein	PITC_037340	2.79	
A0A0A2KR85	Uncharacterized protein	PITC_014180	2.76	
A0A0A2KVZ5	Uncharacterized protein	PITC_028500	2.72	
A0A0A2KMP8	Uncharacterized protein	PITC_017400	2.67	
A0A0A2L522	Mitochondrial carrier protein	PITC_038210	2.51	MF, CC
A0A0A2KX25	Uncharacterized protein	PITC_051360	2.37	
A0A0A2L8T3	Uncharacterized protein	PITC_098640	2.32	
A0A0A2L2N9	HEAT, type 2	PITC_085160	2.10	
A0A0A2L2V7	Acyl-CoA dehydrogenase, N-terminal	PITC_066160	2.10	MF
A0A0A2LCB9	Glycoside hydrolase, family 28	PITC_005000	2.10	BP, MF, CC
A0A0A2LAG1	Xylulose 5-phosphate/Fructose 6-phosphate phosphoketolase, N-terminal	PITC_003850	2.10	BP, MF
A0A0A2L8I5	Glucose-repressible protein Grg1	PITC_061580	2.05	
A0A0A2L934	Polyketide synthase, enoylreductase	PITC_030400	2.04	MF
A0A0A2LBT9	CDR ABC transporter	PITC_091640	0.49	MF, CC
A0A0A2KM09	ATPase, AAA-type, core	PITC_028850	0.48	MF
A0A0A2KZ16	Mandelate racemase/muconate lactonizing enzyme	PITC_098150	0.48	BP, MF
A0A0A2KNF6	Cytochrome P450	PITC_065080	0.47	BP, MF
A0A0A2LD14	Short-chain dehydrogenase/reductase SDR	PITC_006840	0.46	
A0A0A2LAZ8	Uncharacterized protein	PITC_023560	0.45	CC
A0A0A2KLM8	Aldo/keto reductase	PITC_048920	0.43	MF
A0A0A2L925	ABC transporter, integral membrane type 1	PITC_030250	0.41	MF, CC
A0A0A2L7S7	Reverse transcriptase	PITC_011060	0.38	MF
A0A0A2L743	Uncharacterized protein	PITC_032240	0.35	MF
A0A0A2KIE8	Male sterility, NAD-binding	PITC_056110	0.34	MF
A0A0A2LCN6	Short-chain dehydrogenase/reductase SDR	PITC_019130	0.31	
A0A0A2LGP0	Carbon-nitrogen hydrolase	PITC_029870	0.30	BP, MF
A0A0A2KZU8	Peptidase M20	PITC_086250	0.23	MF
A0A0A2LFP8	O-methyltransferase, family 3	PITC_025440	0.21	MF
A0A0A2L830	ThiJ/PfpI	PITC_001680	0.14	
A0A0A2L1V6	N-acyl-phosphatidylethanolamine-hydrolyzing phospholipase D	PITC_051890	0.12	MF
A0A0A2LCF5	Acyl-transferase/acyl-hydrolase/lysophospholipase	PITC_005350	0.08	MF
A0A0A2L5Z5	Uncharacterized protein	PITC_002480	0.08	
A0A0A2L3A1	Uncharacterized protein	PITC_038310	0.07	
A0A0A2LCE5	Uncharacterized protein	PITC_005250	0.06	
A0A0A2LAC2	Uncharacterized protein	PITC_005360	0.04	
A0A0A2LKC5	Monooxygenase, FAD-binding	PITC_005370	0.03	MF, CC
A0A0A2L9W9	Uncharacterized protein	PITC_005390	0.03	BP, MF
A0A0A2KD20	Uncharacterized protein	PITC_008180	0.03	BP, MF
A0A0A2LCH9	Uncharacterized protein	PITC_005280	0.03	
A0A0A2LEG0	E3 ubiquitin protein ligase	PITC_068020	+	BP, MF, CC
A0A0A2KL74	Uncharacterized protein	PITC_064410	+	
A0A0A2L3H1	Kinetochore-Ndc80 subunit Spc24	PITC_084180	+	
A0A0A2LNS4	Zinc finger, RING-type	PITC_024830	+	MF
A0A0A2KNZ0	Translation elongation factor EF1B/ribosomal protein S6	PITC_014590	+	MF, CC
A0A0A2KWT6	Ribosome-releasing factor 2, mitochondrial	MEF2	+	BP, MF, CC
A0A0A2LC54	Peptidase C19, ubiquitin carboxyl-terminal hydrolase 2	PITC_059690	+	BP, MF
A0A0A2LG71	Pectin lyase fold/virulence factor	PITC_050450	+	MF
A0A0A2L5T7	Uncharacterized protein	PITC_001930	+	BP
A0A0A2K9T6	Uncharacterized conserved protein UCP022603	PITC_093160	+	
A0A0A2K9G7	Glycoside hydrolase, superfamily	PITC_049080	+	BP, MF
A0A0A2LF07	CheY-like superfamily	PITC_061050	+	MF
A0A0A2LN85	Taxilin family	PITC_010570	+	MF
A0A0A2KY31	Beta-glucosidase	PITC_028190	+	BP, MF
A0A0A2KE07	Chromatin-remodeling complex, RSC SWI/SNF subunit Rsc7/Swp82	PITC_055960	+	
A0A0A2LB02	RNA polymerase II subunit A	PITC_092370	+	BP, MF, CC
A0A0A2L857	Transcription factor, MADS-box	PITC_001660	+	BP, MF, CC
A0A0A2L6J4	Neurolysin/Thimet oligopeptidase, N-terminal	PITC_084000	+	BP, MF, CC
A0A0A2KT14	Monopolin complex, subunit Csm1/Pcs1	PITC_066000	+	
A0A0A2LE82	Uncharacterized protein	PITC_037500	+	
A0A0A2LBD7	Sof1-like protein	PITC_089150	+	
A0A0A2L5N2	Striatin, N-terminal	PITC_021600	+	
A0A0A2L4U0	tRNA-dihydrouridine(47)synthase [NAD(P)(+)]	PITC_047170	+	MF
A0A0A2L4G7	Phosphatidylserine decarboxylase proenzyme 1, mitochondrial	PSD1	+	BP, MF, CC
A0A0A2KS93	BolA protein	PITC_042910	+	
A0A0A2LEA3	Uncharacterized protein	PITC_034190	+	
A0A0A2KWV6	Sensitivity to Red Light Reduced-like, SRR1	PITC_077860	+	
A0A0A2L4C5	Poly(A) polymerase	PITC_097130	+	BP, MF, CC
A0A0A2L1S8	Heme-containing dehydratase	PITC_040090	+	
A0A0A2L7N4	Winged helix-turn-helix transcription repressor DNA-binding	PITC_006550	+	BP, MF, CC
A0A0A2KI48	Aldolase-type TIM barrel	PITC_009330	+	MF
A0A0A2KWA6	Putative domain, di-copper center	PITC_096580	+	MF
A0A0A2LFQ2	Uncharacterized protein	PITC_025560	+	CC
A0A0A2LAP4	Uncharacterized protein	PITC_036940	+	
A0A0A2KHA2	DNA polymerase III, clamp loader complex, gamma/delta/delta subunit, C-terminal	PITC_071920	+	BP, MF
A0A0A2LFW6	Catalase, mono-functional, heme-containing	PITC_041140	+	BP, MF
A0A0A2L4V0	Sas10/Utp3/C1D	PITC_084500	+	
A0A0A2KMY7	PfkB	PITC_046750	+	
A0A0A2L452	Blastomyces yeast-phase-specific protein	PITC_083580	+	
A0A0A2LFY2	Outer membrane protein, IML2, mitochondrial/Tetratricopeptide repeat protein 39	PITC_041270	+	CC
A0A0A2L4F0	Vacuolar protein sorting-associated protein 28	PITC_032200	+	BP, CC
A0A0A2KNJ7	Ferritin	PITC_095310	+	BP, MF, CC
A0A0A2KCE3	Uncharacterized protein	PITC_093200	+	
A0A0A2L328	CheY-like superfamily	PITC_099880	+	BP, MF
A0A0A2KWF5	Ribosomal protein L53, mitochondrial	PITC_063020	+	CC
A0A0A2LGC1	Uncharacterized protein	PITC_036010	+	
A0A0A2KAJ2	Sec1-like protein	PITC_055020	+	BP, CC
A0A0A2KLU4	H/ACA ribonucleoprotein complex, subunit Nop10	PITC_015880	+	BP, MF
A0A0A2LB80	Uncharacterized protein	PITC_088770	+	MF
A0A0A2L283	Vacuole morphology and inheritance protein 14	PITC_085800	+	BP, CC
A0A0A2LJG8	Uncharacterized protein	PITC_091330	+	
A0A0A2LDT3	Scytalone dehydratase	PITC_055360	+	BP, MF
A0A0A2KPK9	Alpha/gamma-adaptin-binding protein p34	PITC_045290	+	
A0A0A2LF46	Glutamyl-tRNA(Gln) amido-transferase subunit B, mitochondrial	PITC_061230	+	BP, MF, CC
A0A0A2LDQ9	Uncharacterized protein	PITC_055380	+	
A0A0A2KLL9	Acyl-transferase/acyl-hydrolase/lysophospholipase	PITC_048820	+	MF
A0A0A2KPR9	Uncharacterized protein	PITC_054110	+	CC
A0A0A2KYQ5	Magnesium transporter	PITC_061420	+	CC
A0A0A2KM68	Uncharacterized protein	PITC_079240	+	
A0A0A2LE28	Carboxymuconolactone decarboxylase	PITC_044010	+	MF
A0A0A2LBC3	Uncharacterized protein	PITC_016760	+	
A0A0A2LPW9	Uncharacterized protein	PITC_059180	+	
A0A0A2KYR5	Uncharacterized protein	PITC_014450	+	
A0A0A2KPB6	Exon junction complex, Pym	PITC_045720	+	BP
A0A0A2L3C0	Uncharacterized protein	PITC_002320	+	
A0A0A2LDR6	Translation Initiation factor eIF-4e	PITC_055210	+	MF, CC
A0A0A2L3Q9	Uncharacterized protein	PITC_035540	+	
A0A0A2K7Z3	RTA-like protein	PITC_013070	--	CC
A0A0A2K9K0	Uncharacterized protein	PITC_049430	--	CC
A0A0A2KC81	Zinc finger, CCCH-type	PITC_079160	--	MF
A0A0A2KRX9	FAD dependent oxidoreductase	PITC_020210	--	MF
A0A0A2KTM9	AP-3 complex subunit delta	PITC_064680	--	BP, CC
A0A0A2KTQ5	TRAM1-like protein	PITC_000730	--	CC
A0A0A2KY77	MAP kinase	PITC_057580	--	BP, MF, CC
A0A0A2L001	Uncharacterized protein	PITC_075040	--	MF, CC
A0A0A2L1F3	Membrane-associated, eicosanoid/glutathione metabolism protein (MAPEG)	PITC_035700	--	CC
A0A0A2L3U6	Uncharacterized protein	PITC_083660	--	MF
A0A0A2L4J0	RNA recognition motif domain, eukaryote	PITC_097790	--	BP, MF, CC
A0A0A2L843	Serine hydrolase FSH	PITC_001510	--	MF
A0A0A2L8X0	Glutamyl-tRNA synthetase, class Ib, archaeal/eukaryotic cytosolic	PITC_073010	--	BP, MF, CC
A0A0A2L929	Acyl-CoA dehydrogenase, N-terminal	PITC_030080	--	MF
A0A0A2L9E5	Major facilitator superfamily domain, general substrate transporter	PITC_090610	--	BP, CC
A0A0A2LCH4	Major facilitator superfamily domain, general substrate transporter	PITC_004620	--	MF, CC
A0A0A2LEL6	Tetratricopeptide-like helical	PITC_067660	--	MF

^1^ Up-regulated proteins are highlighted in red. Down-regulated proteins are highlighted in green. “+” stands for only detected in treatment group and “--” stands for only detected in the control group. ^2^ BP was biological process, MF was molecular function, CC was cellular component.

**Table 3:** Numbers of DEPs in the treatment group compared with the control group.

Comparisons	Number of Involved Proteins	
	Increased	Decreased
Significantly changing in abundance	19	26
Newly arising/undetectable ^1^	67	17

^1^ The proteins newly arising/undetectable in the treatment group were also treated as significantly up-regulated/down-regulated.

**Table 4:** Pathways encompassed by the DEPs ^1^.

Pathway	Protein Name
Dioxin degradation	Monooxygenase, FAD-binding
Polycyclic aromatic hydrocarbon degradation	Monooxygenase, FAD-binding
Linoleic acid metabolism	Cytochrome P450
Betalain biosynthesis	Putative domain, di-copper center
Sphingolipid metabolism	TRAM1-like protein
Mineral absorption	Ferritin
Longevity regulating pathway	Catalase, mono-functional, heme-containing Translation Initiation factor eIF-4e
Pentose and glucuronate interconversions	Ribulose-phosphate 3-epimerase Glycoside hydrolase, family 28
MAPK signaling pathway - yeast	Transcription factor, MADS-box; Catalase, mono-functional, heme-containing; MAP kinase
mRNA surveillance pathway	RNA polymerase II subunit A; Poly(A) polymerase; Exon junction complex, Pym
Carbon fixation in photosynthetic organisms	Ribulose-phosphate 3-epimerase; Xylulose 5-phosphate/Fructose 6-phosphate phosphoketolase, N-terminal
Pentose phosphate pathway	Ribulose-phosphate 3-epimerase; Xylulose 5-phosphate/Fructose 6-phosphate phosphoketolase, N-terminal
Phenylpropanoid biosynthesis	Beta-glucosidase
Naphthalene degradation	Monooxygenase, FAD-binding
MAPK signaling pathway - plant	Catalase, mono-functional, heme-containing
Chemical carcinogenesis	Membrane-associated, eicosanoid/glutathione metabolism (MAPEG) protein
Mismatch repair	DNA polymerase III, clamp loader complex, gamma/delta/delta subunit, C-terminal
Ferroptosis	Ferritin
Isoquinoline alkaloid biosynthesis	Putative domain, di-copper center
Cyanoamino acid metabolism	Beta-glucosidase
EGFR tyrosine kinase inhibitor resistance	Translation Initiation factor eIF-4e
Drug metabolism - cytochrome P450	Membrane-associated, eicosanoid/glutathione metabolism (MAPEG) protein
Amyotrophic lateral sclerosis (ALS)	Catalase, mono-functional, heme-containing
DNA replication	DNA polymerase III, clamp loader complex, gamma/delta/delta subunit, C-terminal
Metabolism of xenobiotics by cytochrome P450	Membrane-associated, eicosanoid/glutathione metabolism (MAPEG) protein
Melanogenesis	Putative domain, di-copper centre
Platinum drug resistance	Membrane-associated, eicosanoid/glutathione metabolism (MAPEG) protein
Galactose metabolism	Mandelate racemase/muconate lactonizing enzyme
Two-component system	MAP kinase
MAPK signaling pathway - fly	Striatin, N-terminal
Nucleotide excision repair	DNA polymerase III, clamp loader complex, gamma/delta/delta subunit, C-terminal
HIF-1 signaling pathway	Translation Initiation factor eIF-4e
Hepatocellular carcinoma	Membrane-associated, eicosanoid/glutathione metabolism (MAPEG) protein
Lysosome	AP-3 complex subunit delta
FoxO signaling pathway	Catalase, mono-functional, heme-containing
Fluid shear stress and atherosclerosis	Membrane-associated, eicosanoid/glutathione metabolism (MAPEG) protein
Longevity regulating pathway - worm	Catalase, mono-functional, heme-containing
Drug metabolism - other enzymes	Membrane-associated, eicosanoid/glutathione metabolism (MAPEG) protein
Necroptosis	Ferritin
Longevity regulating pathway–multiple species	Catalase, mono-functional, heme-containing
Human T-cell leukemia virus 1 infection	Vacuole morphology and inheritance protein 14
Glutathione metabolism	Membrane-associated, eicosanoid/glutathione metabolism (MAPEG) protein
Tyrosine metabolism	Putative domain, di-copper center
Tryptophan metabolism	Catalase, mono-functional, heme-containing
Peroxisome	Catalase, mono-functional, heme-containing
Glycerophospholipid metabolism	Phosphatidylserine decarboxylase proenzyme 1, mitochondrial
Ribosome biogenesis in eukaryotes	HEAT, type 2,H/ACA ribonucleoprotein complex, subunit Nop10
Insulin signaling pathway	Translation Initiation factor eIF-4e
Starch and sucrose metabolism	Beta-glucosidase
PI3K-Akt signaling pathway	Translation Initiation factor eIF-4e
mTOR signaling pathway	Translation Initiation factor eIF-4e
Viral carcinogenesis	Vacuole morphology and inheritance protein 14
Pathways in cancer	Membrane-associated, eicosanoid/glutathione metabolism (MAPEG) protein
Amino sugar and nucleotide sugar metabolism	Glycoside hydrolase, superfamily
Glyoxylate and dicarboxylate metabolism	Catalase, mono-functional, heme-containing
Aminoacyl-tRNA biosynthesis	Glutamyl-tRNA(Gln) amidotransferase subunit B, mitochondrial
RNA transport	Exon junction complex, Pym; Translation Initiation factor eIF-4e
Autophagy–yeast	Sec1-like protein
Endocytosis	Vacuolar protein sorting-associated protein 28
Spliceosome	Uncharacterized protein

^1^ Up-regulated proteins are highlighted in red. Down-regulated proteins are highlighted in green.

**Table 5:** Comparison of the proteomics result and the PRM result of the three candidate proteins.

Protein Name	Gene Name	Signature Peptides	Proteomics Result ^1^	PRM Result ^2^
Nitro-reductase family protein ^3^	PITC_008180	LGPLLQSHK SISPALWEK	0.03	0.05
Cytochrome P450	PITC_065080	AVLSDAVALVR	0.47	0.35
Monooxygenase	PITC_005370	GALEEALQTYSGVR QLENYDEVDDK SDANTSNAYWNGFHPR	0.03	0.16

^1^ Fold changes of protein abundance in proteomics result. Down-regulated proteins are highlighted in green. ^2^ Fold changes of protein abundance in PRM result. ^3^ Nitroreductase family protein (from *Metarhizium brunneum*, 90.4%).

## Supplementary Materials

Supplementary materials can be found at https://www.mdpi.com/1422-0067/20/14/3459/s1. Table S1. The proteins identified in the treatment group and the control group. Figure S1. Effects of MNQ against *P. italicum* in different concentrations. Figure S2. Top 10 pathway enrichments of the identified DEPs.

## Abbreviations

MNQ	2-Methoxy-1,4-naphthoquinone
MIC	Minimum inhibitory concentration
PRM	Parallel reaction monitoring
FDR	False discovery rate
DEPs	Differentially expressed proteins
GO	Gene ontology
KEGG	Kyoto encyclopedia of genes and genomes
BP	Biological process
CC	Cellular component
MF	Molecular function
ACAD	Acyl-CoA dehydrogenase
ME	Malic enzyme
6PGDH	6-Phosphogluconate dehydrogenase
ROS	Reactive oxygen species
CAT	Catalase
GS	Glutathione synthetase
PPP	Pentose phosphate pathway
RPE	Ribulose-phosphate 3-epimerase
KOs	Protein number at KEGG database
PDA	Potato dextrose agar
PDB	Potato dextrose broth
HCD	Higher energy dissociation
pAGC	Predictive automatic gain control
DMSO	Dimethylsulfoxide
PBS	Phosphate buffered saline
