# Supplementary material for: Label-Free Proteomic Analysis of Molecular Effects of 2-Methoxy-1,4-naphthoquinone on Penicillium italicum
Source: Int J Mol Sci. 2019 Jul 14;20(14):3459. doi: 10.3390/ijms20143459 (PMC6678512; doi:10.3390/ijms20143459)
Supplement: Supplementary file 1 [file ijms-20-03459-s001.zip › Supporting Information/Supporting Information.docx]

Article

Label-free Proteomic Analysis of Molecular Effects of 2-Methoxy-1,4-naphthoquinone on *Penicillium italicum*

Meixia Guo^1^, Xiaoyong Zhang^2^, Meiying Li^1^, Taotao Li^3^, Xuewu Duan^3^, Dandan Zhang^3^, Lianmei Hu^4,*^ and Riming Huang^1,*^

^1^ Guangdong Provincial Key Laboratory of Food Quality and Safety, College of Food Science, South China Agricultural University, Guangzhou 510642, China; GMXWW@stu.scau.edu.cn (M.-X.G.); lmy1982@scau.edu.cn (M.-Y.L.)

^2^ Joint Laboratory of Guangdong Province and Hong Kong Region on Marine Bioresource Conservation and Exploitation, College of Marine Sciences, South China Agricultural University, Guangzhou 510642, China; zhangxiaoyong@scau.edu.cn (X.-Y.Z.)

^3^ Guangdong Provincial Key Laboratory of Applied Botany, South China Botanical Garden, Chinese Academy of Sciences, Guangzhou 510650, China; taotaoli@scbg.ac.cn (T.-T.L.); xwduan@scbg.ac.cn (X.-W.D.); [ddzhang@scbg.ac.cn](mailto:ddzhang@scbg.ac.cn) (D.-D.Z.)

^4^ College of Veterinary Medicine, South China Agricultural University, Guangzhou 510642, China

***** Correspondence: huangriming@scau.edu.cn (R.-M.H.), hulianmei@scau.edu.cn (L.-M.H.); Tel.: +86 20-85283448, +86 20-87571321

Received: date; Accepted: date; Published: date

**Abstract:** *Penicillium italicum* is the principal pathogen causing blue mold of citrus. Searching for novel antifungal agents is an important aspect of the postharvest citrus industry because of the lack of higher effective and low toxic antifungal agents. Herein, The effects of 2-methoxy-1,4-naphthoquinone (MNQ) on *P. italicum* and its mechanism were carried out by a series of methods. MNQ had a significant anti-*P. italicum* effect with a MIC value of 5.0 µg/mL. The label-free protein profiling under different MNQ conditions identified a total of 3037 proteins in control group and treatment group. Among them, there were 129 differentially expressed proteins (DEPs，up-regulated > 2.0-fold or down-regulated < 0.5-fold, p < 0.05), 19 up-regulated proteins, 26 down-regulated proteins, and 67 proteins were specific for the treatment group and another 17 specific for the control group. Of these, 83 proteins were sub-categorized into 23 hierarchically-structured GO classifications. Most of the identified DEPs were involved in molecular function (47%), meanwhile, 27% DEPs were involved in cellular component and 26% DEPs were involved in biological process. Twenty-eight proteins identified for differential metabolic pathways by KEGG were sub-categorized into 60 classifications. Functional characterization by GO and KEGG enrichment results, suggests that the DEPs are mainly related to energy generation (mitochondrial carrier protein, glycoside hydrolase, acyl-CoA dehydrogenase and ribulose-phosphate 3-epimerase), NADPH supply (enolase, pyruvate carboxylase), oxidative stress (catalase, glutathione synthetase), and pentose phosphate pathway (ribulose-phosphate 3-epimerase and xylulose 5-phosphate). Three of the down-regulated proteins selected randomly, nitroreductase family protein, monooxygenase and cytochrome P450 were verified using parallel reaction monitoring. These findings illustrated that MNQ may inhibit *P. italicum* by disruption of metabolic processes, especially in energy metabolism and stimulus response that are both critical for the growth of the fungus. In conclusion, based on the molecular mechanisms, MNQ can be developed as a potential anti-fungi agent against *P. italicum*.

**Keywords:** *Penicillium italicum*; 2-methoxy-1,4-naphthoquinone; proteomics; mechanism; label-free quantitative


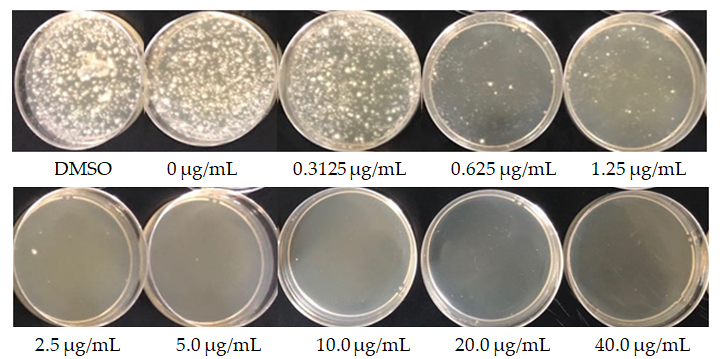


**Figure S1.** Effects of MNQ against *P. italicum* in different concentrations.

**Figure S2.** Top 10 pathway enrichments of the identified DEPs.

| 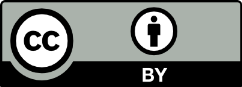 | © 2019 by the authors. Submitted for possible open access publication under the terms and conditions of the Creative Commons Attribution (CC BY) license (http://creativecommons.org/licenses/by/4.0/). |
| --- | --- |
